# From data to practice change – exploring new territory for atlases of clinical variation

**DOI:** 10.1007/s43999-022-00013-3

**Published:** 2022-11-30

**Authors:** Jean-Frederic Levesque, Kim Sutherland

**Affiliations:** 1 Agency for Clinical Innovation, 1 Reserve Road, St Leonards, NSW Australia; 2 Centre for Primary Health Care and Equity, University of New South Wales, Kensington, NSW Australia

**Keywords:** Atlases, Unwarranted clinical variation

## Abstract

Despite decades of atlas production and use within multiple healthcare systems, and consistent reporting of geographical differences in the utilisation of services, significant levels of clinical variation persist. Drawing on over forty years of combined experience using atlases of clinical variation, we reflect on why that might be the case and explore the role of atlases have played in efforts to reduce inappropriate overuse, underuse and misuse of healthcare services. We contend that atlases are useful but, on their own, are not enough to drive change in clinical practice and improvement in patient outcomes. Building on four conceptual models we have published since 2017, we argue that atlases, with their focus on measuring healthcare utilisation by residents in different geographies, generally fail to provide sufficient information and statistical analyses to truly assess the nature of the variation and support action for change. They seldom use structures such as hospitals or teams as the unit of analysis to understand variation; they rarely feature the key elements of healthcare performance which underlie variation; they are mostly silent about how to assess whether the variation measured is warranted or truly unwarranted; nor do they identify evidence-based levers for change. This means that a stark choice confronts producers of atlases – to either continue with the current model and more explicitly rely on other players to undertake work to complete the ‘data to action’ cycle that is necessary to secure improvement; or to refine their offering – including more sophisticated performance measurement approaches, nuanced guides for interpretation of any differences found, support for the selection and application of levers for change that align with local context, and provision of evidence-based options for implementation.

**Supplementary Information:**

The online version contains supplementary material available at 10.1007/s43999-022-00013-3.

## Introduction

Atlases of variation have played an important foundational role in efforts to reduce unwarranted clinical variation across healthcare systems internationally. Primarily illustrating geographic differences in healthcare utilisation, atlases have been produced in multiple jurisdictions over many years [1]. They range widely in terms of breadth and depth of clinical areas covered (Supplementary Table 1) and their approach also features in more focused studies in the peer reviewed literature [2–6]. Despite significant adoption of the atlas approach, evidence demonstrating its impact on actually reducing clinical variation is limited [7, 8]. They are widely regarded as a catalyst for improvement—so necessary but seldom sufficient to secure significant change.

**Fig. 1:**
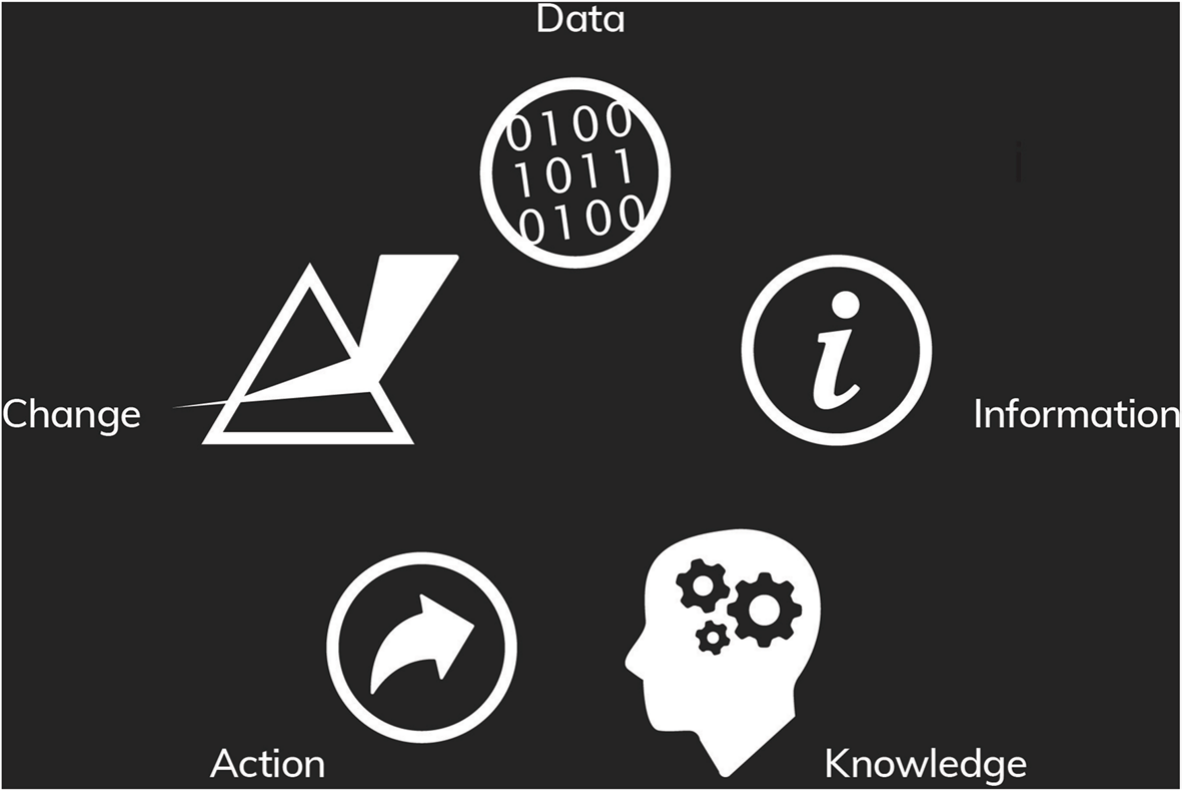
The ‘data to change’ cycle

In this paper, we draw upon our work in healthcare systems on three continents and over the past 20 years, to reflect on the contribution of atlases, their relative strengths, and areas for future development and improvement. Our reflections are informed by managing healthcare organisations focused on measurement, innovation and improvement. This experience has been codified in reports and peer reviewed articles with conceptual models of: performance measurement and reporting [9, 10]; the translation of data and evidence into practice [11–13], and evaluation and change [14]. In this paper we integrate these perspectives and models to reflect on the stages through which data are transformed into information and knowledge, and then translated into action and change (Fig. 1). We situate the ‘data to change’ cycle within an overarching metatheory that encompasses the content or the ‘what’ of change in relation to actors, processes, context and impetus for change.

The data to change cycle is a highly simplified and stylised representation of the complex process by which data is translated into improvements in care. Informed by work in learning healthcare systems [15], environmental studies [16] and data science [17, 18], it comprises five key stages: *from data* which is defined as the codification of physical and theoretical phenomena that can be communicated and analysed; *to information* which is created upon the classification and sorting of data to reveal patterns; *to knowledge* which is the result of interpreting information and testing it with abstract concepts; *to action* with behavioural responses; and meaningful *change*.

Using this cycle as the foundation of our assessment underlines the importance of appraising information products in terms of their capacity to create transformation. It allows us to first, reflect on why healthcare systems are very often rich in data yet remain poor in information and knowledge; second, to highlight that given variation is almost ubiquitous, it is critically important to be able to distinguish noise from signal; and third, to explain why no change is secured when measurement is considered an endpoint.

### The role of atlases in the data to change cycle

While they have been regarded as influential and valuable in bringing to the fore clinical variation as an important issue, in many respects atlases stall at the information stage of the ‘data to change’ cycle. They are necessary but generally not sufficient to achieve meaningful improvement. As a result, there is a growing awareness of the need to build on the foundations laid by atlases to develop more sophisticated and nuanced approaches to measurement, interpretation and action to reduce *unwarranted* clinical variation [19–21].

Atlases are excellent vehicles for displaying utilisation data through a geographical lens – providing valuable information about the scale of differences across and between jurisdictions; but they are ‘not enough’ to fully capture the complexity of healthcare performance [22]. Variation in accessibility, appropriateness, safety and effectiveness of care provided to patients often remains unmeasured yet is crucial in understanding and prioritising the need to change practice and reduce variation.

Multi-construct measurement, while a huge step forward, also needs to be interpreted using conceptual models and frameworks—guiding us to understand what underlies unwarranted clinical variation and where we should focus our efforts to change.

Atlases also need to be conceptualised with clinical engagement and interpretation in mind and provide the required information to identify and engage appropriate levers for change. And knowing which levers to pull is still not enough—the denouement is to *act* – operationalising those levers in ways that are targeted and coordinated. Ultimately, atlases have not to date been conceptualised and produced in ways that enable this action, often using geographies that do not align with the remits of healthcare systems, seldom combining measures that relate to outcomes and resourcing, and rarely providing insights at a level where clinicians can reflect on practice.

In recent years, there are some examples of atlases moving beyond measurement. For example, the Australian Commission on Safety and Quality in Health Care has engaged with clinical groups to better interpret available data and to understand reasons for variation – this work has culminated in a complementary user guide [23]. While this is a welcome development there remains several fundamental issues. Firstly, with metric selection. Atlases are highly focused on a handful of clinical conditions and are yet to resolve how to reconcile the need to be locally applicable and context sensitive and also align with system level priorities and imperatives. They often fall between two stools—too aggregated to support change at a local level yet disconnected from policy and system programs at jurisdictional level. Metric selection cannot be one size fits all. Secondly, questions remain about format – few atlases have harnessed the potential of data science and visualisation to help clinicians explore their practice and how it differs from others; and to help policymakers understand system level issues. For both of these issues, a nuanced approach that accommodates complexity is required.

### Atlases – important but not enough

A range of generic and topic-specific atlases have been produced in Belgium, Canada, England, France, Germany, New Zealand, Spain and the United States [1]. In Australia, the Australian Commission on Safety and Quality in Health Care has since 2015 been publishing atlases of clinical variation (Appendix 1). As is the case in most other jurisdictions, the atlases are primarily populated with utilisation measures –generally reporting variation in the rate per 1000 population of a particular procedure or care episode across geography or socioeconomic groups. Atlases do, on occasion, provide a temporal perspective—revisiting a particular metric to provide data on change over time in the extent of variation seen across geographical areas. However, they rarely explore or account for whether any measured variation is an appropriate reflection of differences in patients’ needs or other contextual issues. Instead, they rely on users to consider whether the scale of the variation reported is unwarranted – something that is far from straightforward to accomplish. The assumption is that a many-fold variation in a clinical practice is likely to be driven by factors other than patients’ needs. A corollary assumption is that many-fold variation between geographic areas, often with many-fold variation in population size and in availability of services, is a reflection of actual clinical decisions.

Although highlighting variation, atlases are often silent on the extent to which the measured variation is in fact unwarranted [24]. Some atlas based assessments do distinguish metrics focused on effective care (where variation implies some underuse of valid treatment), preference‐sensitive care (where variation implies more than one option of care is available and the exercising of patient choice), and supply‐sensitive care (where variation implies the volume of care provided is a reflection of capacity rather than patient need) [22] — however clear distinctions between what is warranted and unwarranted clinical variation remains elusive.

The Australian Commission notes that UCV is “variation that can only be explained by differences in health system performance”.

Based on our previous experience and academic reviews of the field, we define unwarranted clinical variation as *patient care that differs in ways that are not a direct and proportionate response to available evidence; or to the healthcare needs and informed choices of patients* [21].

### What is measurable? The importance of measurement frameworks

**Fig. 2:**
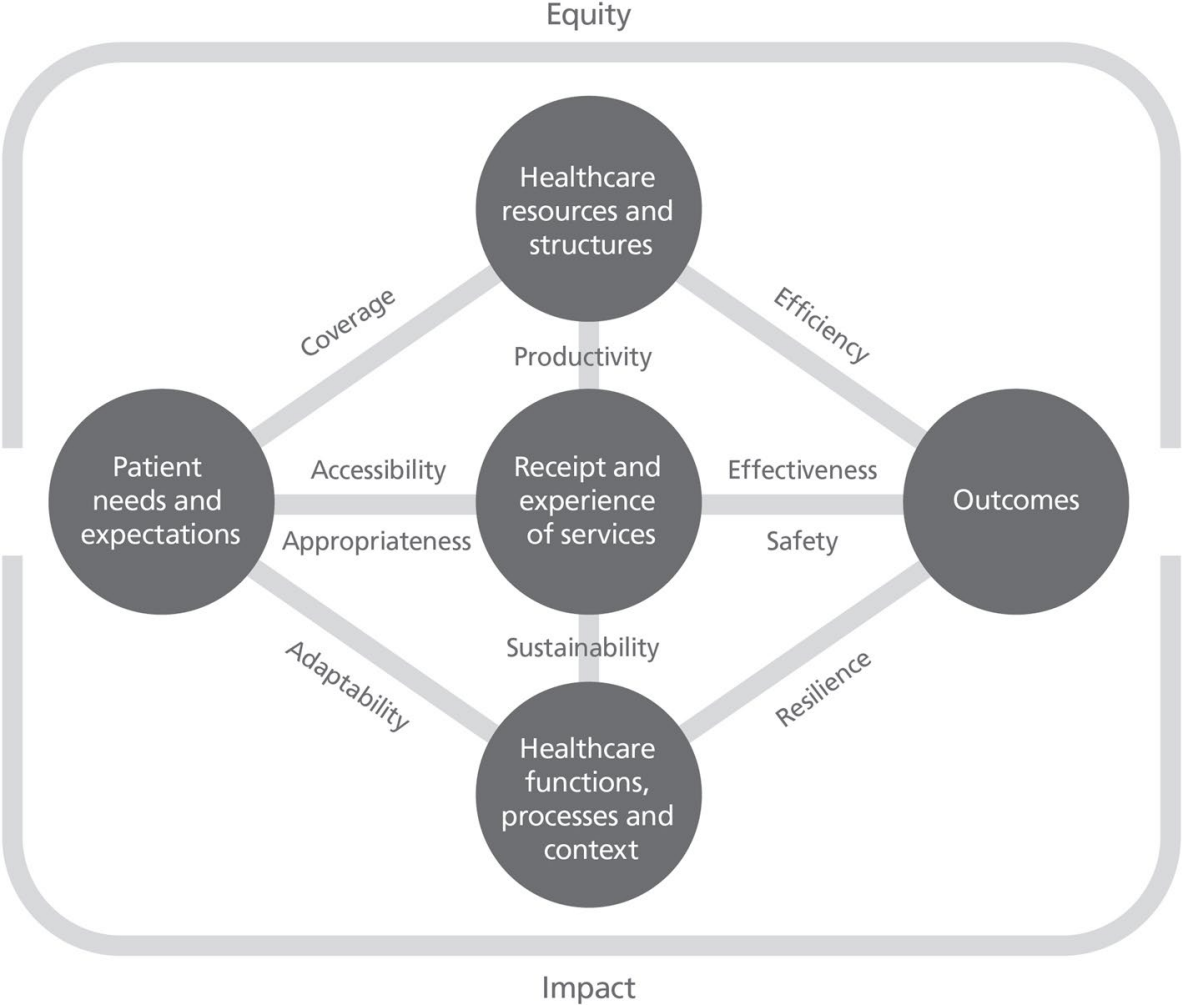
A comprehensive performance framework [10]

Atlases generally focus on metrics that are directly measurable – predominantly receipt of services per capita. Our work on performance measurement has demonstrated that relying on directly measurable metrics – while of value – has considerable limitations in assessing healthcare performance and unwarranted variation therein [10]. It is, of course, of interest to quantify patient needs; or the volume and types of services provided; or outcomes achieved; or resources used; or processes followed. However more nuanced measurement—which uses dynamic or derived metrics formed from two or more of these directly measurable elements—provides much more insight (Fig. 2) [25]. Greater knowledge comes when for example, we consider utilisation relative to patient needs and expectations, or relative to outcomes.

That is because healthcare varies. Variation is ubiquitous. Healthcare responds to each patient’s particular healthcare needs and expectations, social circumstances and capacity to manage his or her own care. Relative or derived metrics help reveal unwarranted variation in constructs such as accessibility, appropriateness, effectiveness and safety – revealing whether patient needs are met with the right type and volumes of services. Was care delivered safely and in a way that maximised potential health gains and patient outcomes?

More sophisticated measurement approaches also allow the focus to move from geography (where someone lives) to providers (who provided the care). It enables fair comparisons between providers, or over time, through adjustment for case mix and other confounders. Ultimately, it may not be variation in utilisation that should be the focus of attention; but variation in the related constructs of accessibility, quality, safety and effectiveness.

There are examples of more comprehensive and nuanced measurement of variation. The Getting It Right First Time (GIRFT) program in England [26] which has been adopted in Queensland Australia, has met with considerable success. Adopting a clinically-led, specialty-focused approach with deep dives into data, supported investigation into underlying reasons for variation and implementation support.

Our experience in various organisations and our academic work in the field of clinical variation, suggests that measuring without a comprehensive framework to interpret the presence and extent of variation, is a risky enterprise. Comprehensive frameworks allow sounder interpretation of the limited number of available measures and enables to assess what measures are missing that would reveal the unwarranted nature or potential causes of variation.

### Measuring routinely collected indicators is not enough – diving deeper

While we argue that atlases would benefit from referring explicitly to comprehensive performance frameworks, we acknowledge that even the most sophisticated measurement framework does not enable us to fully discern unwarranted clinical variation. There is variation across clinical specialties, organisational structures and sectors—different providers offer patients permutations in treatments, clinical pathways and consultations. Healthcare changes over time, with new models of care emerging as new technologies evolve and knowledge develops, is adopted and implemented across a system.

In almost all areas of healthcare where variation has been looked for, it has been found. A systematic review of medical practice variation in OECD countries found 836 published studies, documenting variation across clinical conditions, surgical specialties, and procedures [27].

The last decade has also seen a concerted effort to move away from indicator chaos and towards the judicious use of metrics [28]. With an imperative to address unwarranted clinical variation and the low value care that results from it, we need frameworks to support the interpretation of metrics and allow clinicians, managers and policymakers to see where opportunities for improvement truly lie, and where variation does not have a true impact on care and outcomes.

**Fig. 3:**
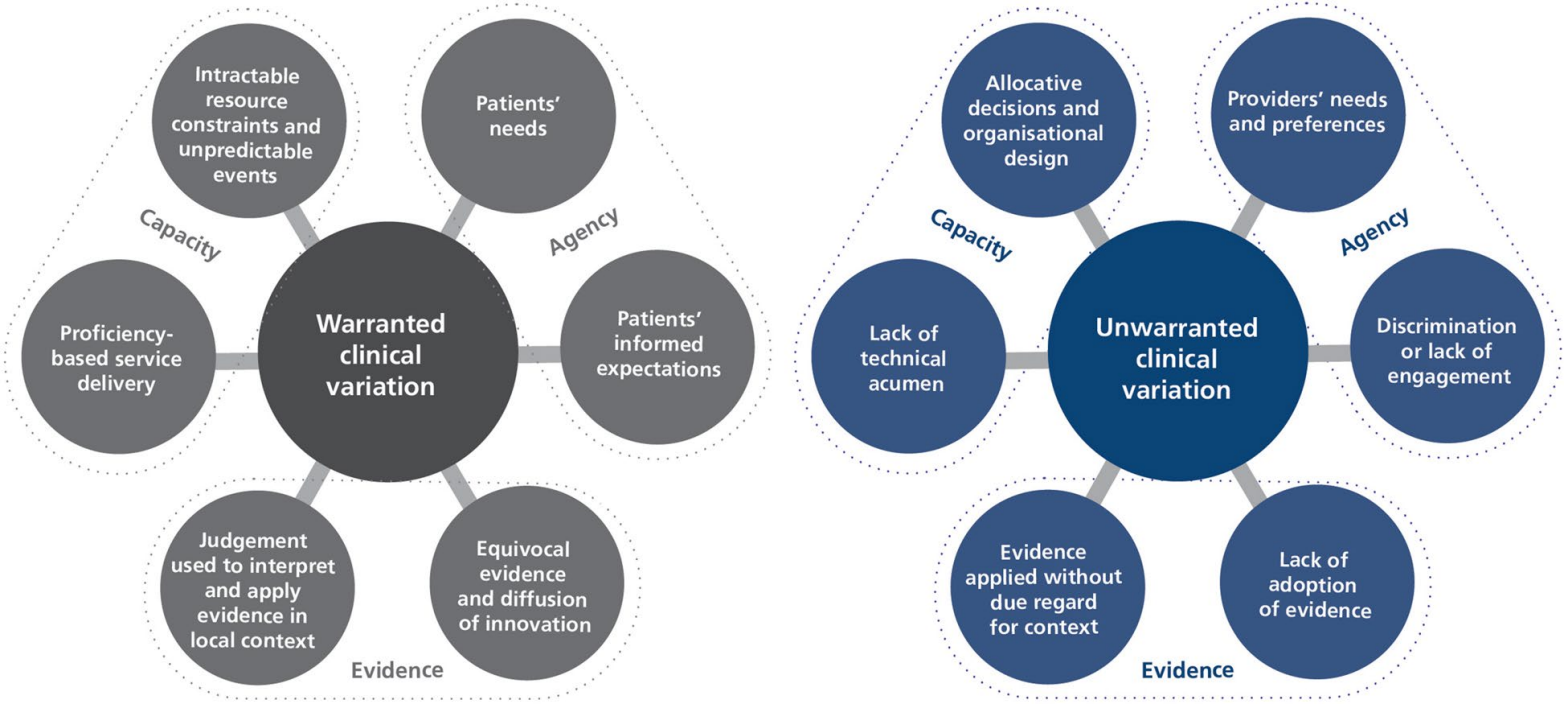
Distinguishing warranted and unwarranted variation [21]

In 2019, we published an analytic framework to help distinguish warranted from unwarranted clinical variation (Fig. 3). It considers whose interests are truly served by provision of treatments, tests and care packages – is it the patient or the provider?; how to interpret differences in patient care in the context of shifting sands of evidence and practice innovation; and whether the nexus of variation is at the level of an individual clinician or a function of fixed organisational constraints [21].

These factors have been used in our experience to challenge the findings from atlases and provide a layer of intelligence over the description of variation contained in atlases. Deliberative processes with consumers and clinicians can be used to provide that critical assessment of variation to better account for equipoise as well as expected delayed diffusion of innovation or adoption of new clinical guidelines in healthcare. We propose that atlases should increasingly aim to integrate these concepts into the selection of indicators to be looked at and the provision of contextual information that will enable this critical assessment of the unwarranted nature of variation. They should also adopt “territories” that are related to the structure of healthcare systems instead of adopting an area of residence approach.

### Identification of unwarranted variation is not enough – what drives variation and behaviour change?

Assessment and categorisation of variation as warranted / unwarranted while essential is not enough– we need to identify appropriate levers for change to address unwarranted variation and its underlying causes in different contexts.

**Fig. 4:**
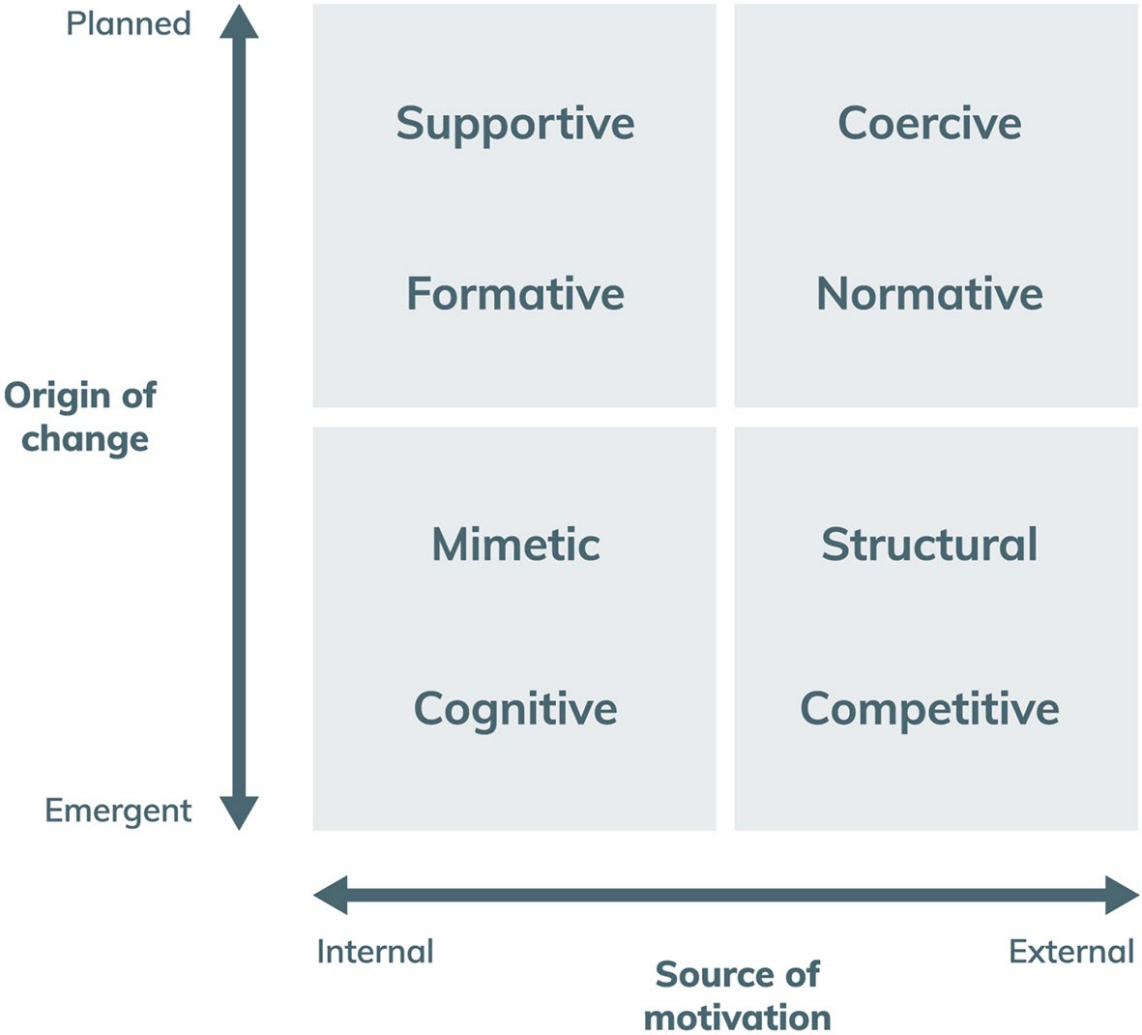
Levers for change [11]

In 2017, we developed a framework to explore levers for change (Fig. 4) [11]. It spans internal and external motivations for change, and emergent and planned processes and identifies eight types of levers: (1) cognitive levers which provide awareness and understanding; (2) mimetic levers to inform about the performance of others to encourage emulation; (3) supportive levers providing implementation tools or models of care to actively support change; (4) formative levers to develop capabilities and skills through teaching, mentoring and feedback; (5) normative levers set performance against guidelines, standards, certification and accreditation processes; (6) coercive levers use policies, regulations incentives and disincentives to force change; (7) structural levers modify the physical environment or professional cultures and routines; (8) competitive levers attract patients or funders.

In complex adaptive systems such as health, a single lever rarely operates successfully in isolation. Meaningful and sustained change is more achievable when different levers work in concert—aligning and reinforcing efforts to improve. For example, normative levers, such as the publication of guidelines, have been shown to have a modest effect on behaviour when applied in isolation however when combined with cognitive, mimetic or coercive levers, they can secure significant reduction in unwarranted clinical variation.

Our recent experience in NSW has demonstrated the contribution that crowdsourcing processes can make in gathering ideas and shape action plans to address unwarranted clinical variation. The implications for atlases of variation is that they need to focus on aspects of care delivery that are influenced by these levers and provide a compendium of measures that will enable the identification of the levers most likely to impact on unwarranted variation. At the moment, atlases focus almost exclusively on cognitive levers: “if you measure it, they will change”.

### What next for atlases of clinical variation?

Reducing unwarranted clinical variation promises to deliver a range of benefits to healthcare systems and to individual patients – enhanced access to care when needed, more reliable provision of indicated and evidence-based care, reduction in wasteful or unnecessary care, improved safety of care, greater system efficiency, and better patient outcomes.

Atlases of clinical variation are an important first step in delivering on this promise – the challenge is to link atlases – which have in many contexts remained siloed – with broader efforts to holistically measure, interpret and change healthcare delivery. Some programs such as GIRFT and the Australian Commission have shown that it is possible to be more comprehensive and integrated in our efforts to tackle unwarranted clinical variation.

**Table:** 

**An example from NSW**
In November 2015, the First Australian Atlas of Healthcare Variation was published [29]. It featured data on stroke average length of stay and noted that of 14,554 admissions for stroke patients aged 65 years and over, the average length of stay ranged from 4.2 to 17.5 days. With no risk adjustment, the extent to which this variation is reflective of case complexity was impossible to assess. We were uninformed about complications, readmissions, mortality and functional outcomes; and about organisational context such as availability of rehabilitation services within the acute hospital setting
Much more meaningful information about unwarranted variation was gleaned from the publication of risk standardised mortality and readmission ratios by the NSW Bureau of Health Information (BHI) [30, 31] – which assessed the extent to which actual patient outcomes varied from those expected, given patients’ age, comorbidities and other factors
The richer information from BHI catalysed efforts to improve – led by the NSW Agency for Clinical Innovation (ACI). Clinicians were engaged in interpreting and explaining variation; and improvement programs and statewide initiatives such as telestroke were developed to enhance access and outcomes for stroke patients throughout the state [32].
In terms of outcomes, 30-day mortality for ischaemic stroke declined by 3.6% since 2015 and the extent of variation reduced
Notably, since the first atlas was published the Australian Commission has developed its approach – and recently published a user guide which outlines ways to explore reasons for measured variation and developing actions to improve [23]

Atlases of clinical variation are at a crossroads. From our perspective, they are important but insufficient to bring about significant improvements in the appropriateness, safety, accessibility and effectiveness of healthcare delivered to patients. We see two clear options for building on the foundations of atlas approaches in the next phase of their development.

The first is for atlases to continue with the current measurement approaches but to be more explicit in passing the baton to other players in healthcare systems to secure passage of data to culminate in action and improvement. They need to be produced at a level of aggregation and with a temporal cycle that enables action, neither too often nor too irregularly. Atlases could be developed and led by clinical groups themselves when mobilisation for action is palpable. Authors of atlases, under this approach, should be very clear about the limitations of their work and remain circumspect about the actual significance of the geographic variation measured.

The second option is for atlases to alter their offering – adopting greater sophistication in measurement approaches, prioritisation of topics where there are real opportunities for significant improvement, nuanced interpretation of the black, the white, and the grey of clinical variation, linkage to levers for change and toolkits for implementation. It is not just about being the first step towards action and change, it is also about measuring in a way that will travel across these subsequent steps and support these stages in a more impactful way. This approach would enable much stronger conclusions to be drawn on the basis of atlases (Table 1).

**Table 1:** Summary of the conceptual models discussed and their potential value for atlases

	Principles	Constructs	Value for atlases
Data to change cycle	Data is translated into improvements in care via a multistage, complex process. It comprises five key stages	• *Data* codification of physical and theoretical phenomena • *Information* created by classification and sorting of data into patterns • *Knowledge* the result of interpreting information and testing it with abstract concepts • *Action* responses to situational knowledge about context and predictions about how acting differently will ultimately result in different outcomes • *Change* Discernible differences in processes, structures or outcomes	Producers of atlases should be explicit about their role in the data to change cycle and how it passes the baton to other organisations and entities
Performance framework [10]	Healthcare performance is a relative construct – it reflects an outcome in relation to a need; a tangible change in relation to context; a benefit in relation to a cost Performance is multi-layered and a contested construct. It is often referred to in terms of ‘value’ or ‘quality’ – notions which can differ across patient, provider, system and population perspectives	• *Coverage* The extent to which services rendered meet the potential need for those services in a community • *Accessibility* The extent to which patients are able: to recognise and identify their healthcare needs; to seek care; to reach providers of care; to pay for care; and to receive care that is proportionate and matched to their needs • *Appropriateness* The extent to which patients receive services that respond to: a) their health needs, b) align with best-practice models of care; c) is delivered in a technically proficient way; d) in accordance with their expectations about the manner in which they should be treated • *Effectiveness* The extent to which healthcare services deliver to patients the benefits expected • *Safety* Whether processes are in place to prevent unnecessary harm to patients –both minimising iatrogenic harm and acting to interrupt patient deterioration and circumvent exacerbations that are amenable to care • *Productivity* The number of goods and services delivered per unit of resource. Often referred to as technical efficiency • *Efficiency* The extent to which healthcare systems and organisations make the best use of available resources. Assessed by quantifying the amount of valued outcomes achieved for the resources invested • *Impact* The influence that services have on a population’s overall health and functioning • *Sustainability* The extent to which healthcare systems function in ways that meet patients’ current health and healthcare needs without compromising the ability to meet needs in the future • *Resilience* At an organisational and system level, resilience is the ability to mount a robust response to unforeseen, unpredicted, and unexpected demands and to resume or continue normal operations • *Adaptability* As the demands for healthcare services—and the technologies available to deliver them—change, systems need to be able to adapt to respond, and planning tools need to recognise the interdependencies within the care service and care infrastructure system • *Equity* The extent to which everyone in a population has the opportunity to reach their full health potential, equity incorporates the idea that receipt of care, appropriateness of care and outcomes of care should be consistent across social groups and responsive to needs	Identifying meaningful variation, the underlying causes of variation, and options for tackling unwarranted variation often requires an approach that moves beyond the directly measurable number of services provided to more nuanced and sophisticated measurement of dynamic performance constructs
Unwarranted Clinical Variation [21]	Tackling unwarranted clinical variation requires a strong conceptual framework to guide rigorous measurement and remediation efforts Agency and motivation, evidence and judgement, and personal and organisational capacity all play key roles in clinical decision making and help distinguish warranted from unwarranted clinical variation	• *Agency and motivation* Encapsulates issues of motivations and for whom clinical decisions are made, focusing particularly on questions about whose needs and expectations drive clinical decisions • *Evidence and judgement* Considers whether clinical decisions align and resonate with the extant knowledge base; the extent of equipoise; the changing nature of available information • *Personal and organisational capacity* Focuses on whether clinicians are able to provide care in the way they seek and includes questions about how decisions are enabled and supported. These relate especially to when variation focuses on clinicians and the need to consider any organisational constraints they face	Once measured, variation requires nuanced interpretation to guide diagnosis and formulate a prescription for change
Levers for change [11]	Change is influenced intrinsic and extrinsic motivators, perceptions of fear or threat and norms, attitudes and intentions Levers are most effective when tailored to context and used in concert	• *cognitive levers* provide awareness and understanding • *mimetic levers* inform about the performance of others to encourage emulation • *supportive levers* implementation tools or models of care to actively support change • *formative levers* develop capability and skills through teaching, mentoring and feedback • *normative levers* set performance against guidelines, standards, certification and accreditation processes • *coercive levers* policies, regulations incentives and disincentives to force change • *structural levers* modify the physical environment or professional cultures and routines • *competitive levers* use market forces to attract patients or funders	*Knowing is not enough- we must do* (Goethe) – understanding how information from atlases will be used to leverage change is key to success

Having reflected upon the use of atlases and how they could be informed and strengthened in the future, we contend that the adoption of an integrated, intelligent approach to measurement which explicitly considers how to support the ‘data to action’ cycle proffers huge potential gains in value and quality in healthcare. However, embarking upon a program that more fully considers various constructs that relate to performance measures, and the potential causes of unwarranted variation, may seem overly ambitious and promoters of atlases may want to keep their approach pragmatic and simple. But the easier solution risks remaining an ineffective solution in the future. In the context where advances in data generation (e.g. use of trackers in healthcare, data derived from the use of electronic records, data generated through internet of things) and in data processing capability (e.g. artificial intelligence and machine enhanced learning, data analysis automation, powerful business intelligence tools), atlases of variation should evolve and harness these new technologies. Strong conceptualisations to guide this and approaches that more directly support the processes of healthcare change will be required.

### Supplementary Information

**Additional file 1: Supplementary Table 1.** A selection of Atlases of Clinical Variation published in developed healthcare systems.

## Data Availability

Supplementary tables have been provided and appropriate reference made to original work cited in this work. There are no data collected as part of this work.
