# Supplementary material for: From data to practice change – exploring new territory for atlases of clinical variation
Source: Res Health Serv Reg. 2022 Nov 30;1:13. doi: 10.1007/s43999-022-00013-3 (PMC11281751; doi:10.1007/s43999-022-00013-3)
Supplement: Supplementary file 1 — Additional file 1: Supplementary Table 1. A selection of Atlases of Clinical Variation published in developed healthcare systems. [file 43999_2022_13_MOESM1_ESM.docx]

Supplementary Table 1

**A selection of Atlases of Clinical Variation published in developed healthcare systems**

| Jurisdiction | Years; | Topics |
| --- | --- | --- |
| Australia | 2015  2017  2018  2021 | Antimicrobial dispensing  Diagnostic interventions (fibre optic colonoscopy; prostate biopsies 40+ years; CT of lumbar spine; gastroscopy; thyroid investigations; cardiac tests)  Surgical interventions (knee arthroscopy 55+ years; cataract 40+years; lumbar spine surgery; radical prostatectomy; hysterectomy and endometrial ablation; tonsillectomy; myringotomy; hip fracture; knee replacement; lumbar spinal decompression; lumbar spinal fusion; laparascopic cholecystectomy; appendicectomy; thyroidectomy)  Interventions for mental health and psychotropic medicines (GP mental health treatment plans; antidepressant / anxiolytic / antipsychotic / ADHD medicine dispensing)  Opioid medicine dispensing  Proton pump inhibitor dispensing  Medicine use in older people  Interventions for chronic disease (asthma and COPD medicine dispensing and admissions; heart failure admissions; diabetes lower limb amputation; stroke ALOS; anticholinesterase medicine dispensing)  Potentially preventable hospitalisations (COPD, heart failure, cellulitis, kidney and urinary tract infections, diabetes complications)  Cardiovascular conditions (AMI admissions, atrial fibrillation)  Women’s health and maternity (Caesarean section, 3^rd^ and 4^th^ degree perineal tears; early planned caesarean section) |
| Belgium | 2006 | Hip replacement, knee replacement, knee arthroscopy, carpal tunnel surgery, cataract surgery, carotid artery surgery, hysterectomy, C-section.  Bariatric surgery, myringotomy, phlebotomy, medical imaging (MR,CT), appendectomy, tonsillectomy, knee and hip replacement. |
| England | 2010  2011  Thematic since 2012 | Around 100 indicators are mapped in the 2016 compendium Atlas (up from 34 in the first edition). |
| France | 2016 | Tonsillectomy, appendectomy, C-section, bariatric surgery, prostate surgery, carpal tunnel surgery, cholecystectomy, hysterectomy, knee replacement, thyroidectomy. Hip fracture surgery |
| Spain | Since 2006 | Orthopaedic surgery (hip fracture, knee and hip replacement), general surgery, paediatric hospitalisations, cardiovascular procedures (including stroke management), diabetes care, cancer care, hospitalisations for mental health problems, avoidable hospitalisations for frail patients or chronic conditions and procedures considered lower-value care |
| US | Dartmouth Atlases Since 2005 | Cardiac surgery; AAA repair; spinal surgery; primary care; end of life care; carotid revascularisation; hip, knee and shoulder replacement; Medicare spending; post-acute care; prescription drug use; children’s health care; bariatric surgery; diabetes and peripheral arterial disease; end-stage renal disease; neonatal intensive care; |
